# Challenges and opportunities in perinatal public health: the utility of perinatal health inequality dashboards in addressing disparities in maternal and neonatal outcomes

**DOI:** 10.1186/s12884-024-07056-z

**Published:** 2024-12-20

**Authors:** Olufisayo Olakotan, Jennifer N. W. Lim, Thillagavathie Pillay

**Affiliations:** 1 Department of Neonatology, Women and Children’s Directorate, University Hospitals Leicester NHS Trust, Leicester, UK; 2 Faculty of Education, Health and Wellbeing, University of Wolverhampton, Wolverhampton, UK; 3 Faculty of Science and Engineering, University of Wolverhampton, Wolverhampton, UK; 4 Department of Population Health Sciences, University of Leicester, Leicester, UK; 5 University Hospitals Leicester, Neonatal Unit, Leicester Royal Infirmary, Leicester, LE1 5WW UK

**Keywords:** Perinatal, Maternal, Neonatal, Health inequality, Equity, Disparities

## Abstract

**Introduction:**

In clinical settings, digital dashboards display medical data, with the aim of identifying trends and signals. In so doing these contribute towards improving service delivery and care within hospitals. It is not clear whether the utility of perinatal health equity dashboards could be used to identify health inequality trends that could potentially impact on health service delivery, care and public health interventions. This study aims to evaluate the implementation of health inequality dashboards that address disparities in maternal and neonatal outcomes, with a specific focus on identifying key challenges encountered during their deployment and use in healthcare settings.

**Methods:**

Three databases, namely Embase, CINAHL, and Medline were searched to identify relevant studies in English Language published between 2010 and 2022. All findings were reported according to PRISMA guidelines for scoping reviews.

**Results:**

Of 670 identified articles, only 13 met the inclusion criteria. The study identified three key themes: dashboard functionality, data accuracy, and challenges in collecting health inequality data. Dashboards were used to visualize disparities, with functionalities focusing on specific audiences, contents, and utility. Issues with data completeness, standardization, and challenges in collecting consistent health inequality data, especially from diverse ethnic groups, hindered the accurate tracking of maternal and neonatal disparities.

**Conclusion:**

The use of perinatal health inequality dashboards is a critical step forward in optimizing maternal and neonatal care by providing targeted interventions. However, further research is needed to assess their long-term impact on reducing health inequalities, while addressing challenges related to data accuracy, completeness, and standardization to improve their effectiveness.

**Supplementary Information:**

The online version contains supplementary material available at 10.1186/s12884-024-07056-z.

## Introduction

Significant disparities in perinatal outcomes exist globally, disproportionately affecting the most vulnerable and socio-economically disadvantaged populations, often from ethnic minority groups, a phenomenon known as health inequality [1–3]. Socio-economic deprivation among ethnic minority groups limits access to quality healthcare, nutrition, and safe living conditions. When combined with additional barriers such as systemic discrimination, language differences, and cultural misunderstandings, this further reduces the quality of care during pregnancy and contributes to disparities in health outcomes [4–6]. In the United Kingdom (UK), the National MBRRACE (Mothers and Babies: Reducing Risk through Confidential Enquiries) data highlights significant disparities in perinatal outcomes among racial and ethnic minorities [7, 8]. Between 2018 and 2020, maternal mortality rates were 3.7 times higher for Black women and 1.8 times higher for Asian women compared to their white counterparts in the UK [9]. Similar trends are observed in other developed economies. For example, in the United States, Canada, and Australia, Black mothers not only face higher maternal mortality rates than white mothers, but they also give birth in hospitals with higher risk-adjusted rates of stillbirth and neonatal death [5, 10–13].

Despite the recognition of these disparities, less attention has been given to the use of quality improvement tools in addressing them. One emerging solution is the use of health inequality dashboards, which enable the visualization and analysis of health data to identify disparities and facilitate targeted interventions [14–16]. For example, an interactive dashboard developed to track Covid-19 trends and racial inequalities across US cities allowed practitioners to compare disparities and identify structural factors such as racism, segregation, and socio-economic conditions affecting health inequalities in urban areas [14]. In England, an inequality dashboard was developed to track healthcare inequalities in deprived neighbourhoods, using eight indicators, including patient-to-GP ratios and primary care quality, for areas with populations over 100,000 [15]. The dashboard visually presented data to help decision-makers address disparities.

Similarly, in the United States, a paediatric inequality dashboard was developed to track healthcare disparities, highlighting performance trends, missed opportunities, and disparities in outcomes between Black/African American and white patients, with a focus on quality improvements [16]. While these dashboards show promise, there remains limited research on their effectiveness in improving maternal and neonatal care outcomes, especially for disadvantaged populations [14–16]. This review fills a gap by synthesizing findings from the literature on evaluating the functionalities, challenges, and potential uses of dashboards in healthcare settings, with a secondary mention of their impact on outcomes. This study aims to evaluate the implementation of health inequality dashboards that address disparities in maternal and neonatal outcomes, with a specific focus on identifying key challenges encountered during their deployment and use in healthcare settings.

## Methods

### Information sources and Search strategy

This scoping review was conducted in accordance with the PRISMA Extension for Scoping Reviews (PRISMA-ScR) checklist (Appendix C). We conducted a search of Medline, Embase, and CINAHL comprehensive sources of health-related literature, particularly focusing on maternal and neonatal care between November 1, 2022, and December 15, 2022. Using the relevant keywords shown in appendix B, we covered the period from 2010 to 2022. The time frame was chosen based on our database search, as research on perinatal health inequality dashboards in maternal and neonatal care began to emerge around 2010. We set 2022 as the endpoint because that is when the search was conducted. In addition to the selected databases, we also searched the World Health Organization (WHO) Mortality Database and performed forward and backward citation searches on the included articles to identify any additional relevant studies.

### Study selection criteria

#### Inclusion criteria

We established the inclusion criteria for the review using the PICOS framework, which included population, intervention, comparison, outcome, and study design.

*Population*: We included studies that examined maternal and neonatal outcomes in any group of people, regardless of their characteristics such as age, gender, race, ethnicity, or geographic location to capture a comprehensive range of health disparities. While we recognize that this broad inclusion criterion may introduce heterogeneity, it was necessary to identify overarching trends in health inequalities. We addressed potential heterogeneity by carefully synthesizing the findings, focusing on themes that explore how health inequality dashboards are used to monitor disparities in maternal and neonatal care.

*Intervention*: we included any study that assess the effectiveness of using health inequality dashboards to highlights or reduce disparities in maternal and neonatal health outcomes.

*Comparison*: we included the utility of health inequality dashboards and other types of data visualization tools that addresses disparities in maternal and neonatal care or other health outcomes such as paediatric care.

*Outcomes*: we considered the outcome of interest as the impact of health inequality dashboards on maternal and neonatal outcomes. Outcomes measured included access to maternal care, maternal mortality rates, infant mortality rates, neonatal morbidity rates, and other indicators of maternal and neonatal health and well-being.

*Study designs*: We considered all study designs such as retrospective and prospective cohort studies, mixed method, and reports due to the limited studies published in this area.

#### Exclusion criteria

We excluded studies that examined disparities in maternal and neonatal outcomes without addressing the use of health inequality dashboards or other visualization tools to mitigate these disparities [17–22]. Additionally, studies focused solely on the technical development of dashboards such as software design, user interface creation, or data visualization techniques were excluded if they did not explore their implementation, use, or associated challenges [23–30]. Articles unrelated to maternal and neonatal outcomes were also excluded, including those addressing quality improvement initiatives, socioeconomic factors, technological advancements, racial bias, telehealth, or other health interventions [31–34] etc., were excluded (Appendix D).

### Screening of articles

All articles retrieved from databases were imported into RefWorks, a reference management tool chosen for its ability to efficiently organize large numbers of references and handle duplicate removal. To guide the screening process beyond RefWorks, we used Rayyan, a web-based tool designed for reviews to screen the titles and abstracts. Titles and abstracts were screened independently by two reviewers (OO, TP). Conflicting decisions were resolved through discussion, leading to modification of inclusion and exclusion criteria. For the final selection of full-text articles, three reviewers (OO, TP, JNL) met virtually to reach a consensus.

### Data extraction

We used a standardized data extraction form to extract the following types of data: study population characteristics, dashboard designs, methodology, maternal and neonatal outcomes measured, and key findings. A quality assessment of the selected articles was not conducted. Excluding the study quality assessment allowed us to capture studies that offer valuable insights despite methodological variations.

### Data analysis and synthesis

We performed a thematic analysis to identify key themes across the included studies. The themes emerged inductively, based on the data extracted from the studies. Two reviewers (OO, JNWL) identified recurring concepts and patterns from the data, which were grouped into themes, synthesized the findings to present them clearly and concisely.

## Results

### Study selection

**Fig. 1:**
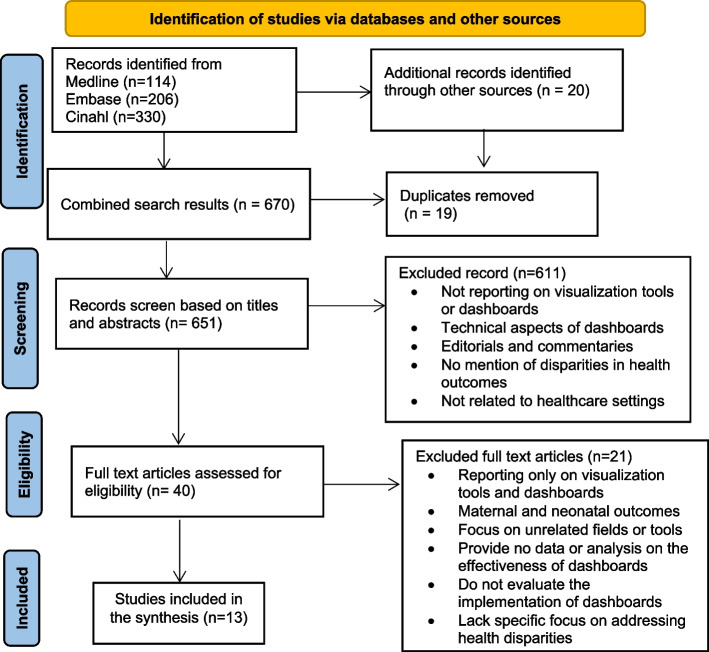
Study selection

The database search returned 670 results. After removing duplicates, 651 titles and abstracts were reviewed, narrowing the selection to 40 articles for full-text review (Fig. 1).

During the full-text review, more stringent criteria were applied to ensure that the articles directly linked health inequality dashboards to maternal and neonatal outcomes. As a result, 21 studies were excluded, and only 13 studies were included in the final synthesis (Fig. 1).

### Overview of study designs

The summaries of the thirteen articles included in the review are shown in Appendix A below. Seven studies were conducted in the US, one in Italy, one in Canada, one cross-national study across eight European countries, and three in the UK and Ireland. Two studies employed user-centred design to tailor dashboards to user needs [35, 36]. Four used retrospective data analysis to inform dashboard development based on maternal and neonatal outcomes [2, 16, 37, 38]. Two applied cross-sectional analysis to identify real-time inequalities for immediate dashboard translation [39, 40]. One used a case study and stakeholder engagement to design cultural appropriate dashboards [41], while another utilized multistate quality improvement initiative to standardize health data across regions, ensuring consistency in dashboard metrics [42]. One descriptive population-based study analysed maternal mortality across eight European countries, offering comprehensive data to inform dashboard development [43]. Finally, two studies relied on surveillance data and reviews of maternal deaths to understand maternal and neonatal outcomes, providing critical insights into health inequalities [7, 9]. These study designs offer a comprehensive approach by incorporating real-time data and standardized metrics to inform dashboard development, providing valuable insights into how dashboards can effectively address disparities in maternal and neonatal health outcomes.

### Study findings

The study identified key themes, including dashboard functionality, data accuracy, and challenges in collecting health inequality data, which are discussed below.

### Dashboard functionality

The dashboards varied in their functionality, and to better understand their impact, their functionalities were categorized based on three dimensions: audience, content, and utility, highlighting their effectiveness in addressing inequalities in maternal and neonatal outcomes.

### Audience

The primary users of these dashboards include policymakers, public health leaders, and healthcare providers. For example, the national MBRRACE-UK perinatal surveillance dashboard provides healthcare providers with interactive charts that track trends in mortality rates over time [44]. The Ohio Children’s Opportunity Index (OCOI) dashboard, on the other hand, helps policymakers and public health leaders make informed decisions to improve children’s well-being and promote health inequality [35]. Additionally, the maternal-newborn dashboard targets healthcare providers, guiding them in improving performance and patient outcomes [38].

### Content

The OCOI dashboard organizes data into fifty-three measures across eight categories: family stability, infant health, children’s health, access to resources, education, housing, environment, and criminal justice, providing insights into the well-being of infants and children [35]. Similarly, the Opportunity Index Dashboards (OID) provide data on area deprivation indices (AID) and offer valuable information on Black mothers and other ethnic minority women facing multiple risk factors, such as anaemia and hypertension, before, during, and after pregnancy [36]. The maternal-newborn dashboard offers feedback on key performance indicators (KPIs) related to process and outcome measures, providing real-time comparisons with established benchmarks [38].

### Utility

These dashboards serve various purposes, including raising awareness, supporting decision-making, and facilitating quality improvement. The OCOI dashboard identifies social determinants of health (SDOH) challenges and drives actions to improve health inequality for children in Ohio [35]. Similarly, the OID dashboard informs strategies to reduce infant mortality and health disparities, particularly among Black mothers and ethnic minorities [36]. The maternal and neonatal dashboards focus on driving quality improvement by enhancing awareness of KPIs and offering real-time feedback to healthcare providers [38].

### Accuracy of data

Ensuring accuracy of data in the EHR, such as SDOH, race, and ethnicity data, is crucial for targeting interventions, and inform the development of perinatal health inequality dashboards [45]. However, limitations related to data completeness and standardization impact the usefulness of health inequality dashboards.

### Data completeness

Sun et al. (2022) reported that Black patients had 2.54 times the odds of having at least one negative descriptor in the history and physical notes of their EHR compared to White patients, even after controlling for sociodemographic and health characteristics, suggesting systemic racial bias in how patients are documented in medical records [40]. Similarly, Wark et-al. (2022) found that outdated SDOH data in EHR systems hindered healthcare providers’ ability to access real-time information, making it challenging to deliver targeted interventions to those in need [41]. In line with these findings, Romero et.al. (2022) through a Multistate Data Strategy, reported that key SDOH data, such as employment and economic stability critical for understanding social risk factors contributing to COVID-19 morbidity and disparities in infection rates among different population groups were often missing from the EHR [42].

### Data standardization

Studies have identified major problems with EHR systems, particularly due to the absence of structured SDOH data variables and tags. For example, one study reported that the lack of standardized fields to categorize SDOH in EHR data has made it difficult to collect, analyse, and utilize information across various health systems [41]. Similarly, Romero et.al (2022) noted that the use of federally mandated terminology mappings, such as Logical Observation Identifiers Names and Codes (LOINC) and Prescription for Electronic Drug Information Exchange (PEDIE), hindered data quality and extraction efforts [42]. This underscores the need for standardized, structured EHR data formats to effectively capture and utilize relevant SDOH information [41, 42].

### Challenges in collecting health inequality data

The difficulties in collecting health inequality data in maternal and neonatal care, particularly from diverse ethnic groups, pose a barrier to the effective use of dashboards in addressing maternal and neonatal outcomes, as these dashboards rely heavily on data quality. For instance, the Italian Obstetric Surveillance System (ITOSS) was developed to address data gaps by leveraging incident case reporting and confidential inquiries. While ITOSS improves data completeness, it also underscores the broader challenge of obtaining comprehensive data necessary for accurate dashboard analysis [2]. Similarly, a study on maternal mortality across eight European countries, including France, Denmark, and the UK, used enhanced surveillance systems to streamline the identification and documentation of maternal deaths [43]. Despite these efforts, cross-national comparisons of maternal ethnicity data against national vital statistics registers reveal inconsistencies in data collection methods, limiting the ability of dashboards to provide standardized insights across different contexts.

Challenges in data surveillance further complicate dashboard effectiveness. The MBRRACE-UK "Confidential Enquiry into Maternal Deaths and Morbidity" report, for example, captures essential demographic and clinical details but highlights issues with data completeness when cross-referenced with national databases [7, 9]. These gaps compromise the dashboards’ ability to accurately reflect disparities and prioritize interventions. Similarly, the National Perinatal Information Centre (NPIC) report in the US, which shapes race and ethnicity dashboards, illustrates the difficulty in creating comprehensive tools to address health disparities due to inconsistencies in data collection [45].

The Pregnancy Risk Assessment Monitoring System (PRAMS) managed by the US Centres for Disease Control and Prevention (CDC) faces similar challenges [39, 46]. PRAMS collects data on maternal behaviours and experiences across different states but struggles with standardization and completeness. These challenges impact the dashboards’ capacity to inform effective policy decisions and targeted interventions aimed at reducing maternal and infant morbidity [39, 46]. Without addressing these foundational data collection issues, the dashboards’ potential to drive health equity remains constrained.

## Discussion

Current research in maternal and neonatal care highlights the need for a pragmatic approach to reducing health inequality among disadvantaged pregnant women, as racial, ethnic, and socioeconomic disparities remain a significant concern for healthcare researchers [47, 48]. Health inequality dashboards have emerged as valuable tools in addressing disparities in maternal and neonatal care. This discussion examines their benefits, challenges, and limitations in improving maternal and neonatal outcomes.

### Benefits

A qualitative study found that a disparities and equity dashboard in neonatal intensive care units (NICUs) could influence unit-specific practices, motivate staff, address unmeasured inequities, and inform policy on a statewide and national level [49]. Other research has shown that health inequality dashboards provide a national benchmark for hospitals to track key metrics and identify disparities in maternal and neonatal care, particularly related to race and ethnicity [50]. While the benefits of these dashboards cannot be overstated, their thoughtful deployment in healthcare settings is essential to avoid unintended consequences, such as reinforcing stereotypes and group labels. Although dashboards are emerging as a valuable tool for identifying disparities, they have limitations in capturing interpersonal and structural racism [49].

Our review also highlights that SDOH, which encompass the environmental and social conditions in which people live, work, and play, are crucial to consider when introducing interventions like dashboards. For instance, a study in Zimbabwe demonstrated that visualizing adverse birth outcomes on a maternal mortality dashboard gave healthcare providers valuable insights into patients’ SDOH, helping policymakers make informed decisions to reduce maternal and infant mortality across communities [51]. Another study conducted at a public hospital in Zimbabwe found that interactively visualizing adverse birth outcomes on a maternal mortality dashboard provided healthcare providers with valuable insights into patients’ social determinants of health (SDOH), helping to identify individuals likely to experience multiple risk factors before, during, and after pregnancy [52].

### Challenges

While health inequality dashboards offer significant benefits, several challenges remain, particularly regarding data accuracy and timeliness. For example, data from England between 2015 and 2017 indicated that socioeconomic inequality contributed to 24% of stillbirths, 19% of preterm births, and 31% of cases of fetal growth restriction [9]. Adjusting for factors such as ethnicity significantly reduced these percentages, highlighting the critical need for accurate data [9]. Additionally, outdated government policies, like redlining in the U.S., continue to adversely affect communities of colour, resulting in skewed data [53, 54]. Redlined neighbourhoods exhibit higher rates of preterm births compared to better-graded areas, underscoring the impact of structural racism [53, 54]. These disparities complicate data collection and interpretation, ultimately limiting the effectiveness of health inequality dashboards.

In addition, the availability of neonatal data remains a significant challenge. For example, in 2015, birthweight data was unavailable for approximately 39.7 million newborns globally, with Africa accounting for more than half of these cases [55]. These data gaps hinder the development of comprehensive dashboards. Improving national surveillance systems can enhance data collection and reporting on birth outcomes, such as low birthweight and preterm birth, and enable governments to set targets, develop effective dashboards, and monitor interventions over time [55].

Ethical concerns, particularly around data privacy and security, pose significant challenges for health inequality dashboards [56, 57]. These tools rely on sensitive information, such as race, ethnicity, and socioeconomic data for high-risk populations, which may lead to discrimination or stigmatization, making it critical to protect patient confidentiality [58]. To mitigate these risks, data anonymization and encryption techniques should be implemented, as demonstrated in the Rakai Community Cohort Study, where privacy risks are mitigated, while dashboards continue to provide valuable insights into health inequalities [56]. Additionally, transparency regarding data usage and informed consent is essential to maintaining trust, ensuring that individuals are aware of how their data is used [58]. Addressing these ethical concerns is vital to ensuring dashboards are both secure and effective in reducing health inequalities.

### Limitations

Although dashboards offer valuable insights, there are limitations to their implementation and effectiveness. Measuring the actual impact of dashboards in addressing health inequality remains challenging. To address this challenge, potential methods for evaluating the effectiveness of dashboards could include short-term metrics such as the timeliness of data reporting, improving engagement with healthcare stakeholders, and integrating data on disparities in health outcomes into electronic health records (EHRs) to better inform decision-making processes [59, 60]. Additionally, quantitative indicators such as patient wait times, readmission rates, and changes in health outcomes over time across different socio-economic groups could provide a more tangible link between dashboard use and improvements in health inequality [61, 62].

### Study limitations

The limitations of this study include the small number of published studies on health inequality dashboards in maternal and neonatal care, which makes it difficult to assess the usefulness of these dashboards across various populations, groups, or healthcare settings. This limitation affects the robustness of our findings and may introduce bias, as the studies reviewed may not be representative of broader healthcare systems or underserved populations or groups. To address these limitations, more longitudinal research is needed to evaluate the long-term impact of dashboards, which will help provide a more comprehensive understanding of their role in improving maternal and neonatal care across different healthcare settings.

## Conclusion

This review highlights the significant role health inequality dashboards play in addressing disparities in maternal and neonatal outcomes. Their utility ranges from raising awareness to facilitating decision-making and improving quality of care. However, the effectiveness of these dashboards is contingent upon the accuracy, timeliness, completeness, and standardization of data, which are significant limitations in their current use. To address the identified limitation, improving data timeliness through the adoption of real-time data entry systems in EHRs, particularly for SDOH, race, and ethnicity variables, would enhance the relevance and accuracy of dashboards. Additionally, future research should focus on integrating qualitative data alongside quantitative metrics to provide deeper insights into the lived experiences of underserved populations. This approach would enrich the dashboards with contextual information that may be missing from EHRs, ensuring a more holistic understanding of health inequalities.

## Supplementary Information

Supplementary Material 1.
