# Supplementary material for: Challenges and opportunities in perinatal public health: the utility of perinatal health inequality dashboards in addressing disparities in maternal and neonatal outcomes
Source: BMC Pregnancy Childbirth. 2024 Dec 20;24:837. doi: 10.1186/s12884-024-07056-z (PMC11660982; doi:10.1186/s12884-024-07056-z)
Supplement: Supplementary file 1 — Supplementary Material 1. [file 12884_2024_7056_MOESM1_ESM.pdf]

Appendix A. Characteristics of selected studies

Author, year, country	Study population characteristics	Brief description of methodology	Dashboard designs	Maternal and neonatal outcomes measured	Key findings
Pallavi Jonnalagadda et-al 2022, United States (US) [17],	The study focuses on children in Ohio (ages 0-17), accounting for 2.6 million of ONAhio's 11.5 million population.	User-centred design approach to developing the two OCOI dashboards	The Ohio Children's Opportunity Index (OCOI) includes two dashboards	infant mortality, preterm births, maternal morbidity,	The OCOI dashboard identifies census tracts with multiple adverse health indicators, revealing significant disparities in health outcomes. Areas with lower opportunity scores, often with higher minority populations, show worse outcomes, particularly for infant health.
Naleef Fareed et-al 2020, US [18]	Focus on Ohio census tracts, highlighting disparities in infant mortality, especially among Black infants (15.6 per 1,000 vs. 5.3 for White infants)	User-centred design approach involving feedback sessions with stakeholders, who included representatives from project sponsors and subject matter experts.	The Ohio Opportunity Index (OOI) Dashboard, built in Tableau, visualizes seven domains: transportation, education, employment, housing, health, access to services, and crime etc.	Infant mortality, preterm births, maternal health conditions, and environmental risks	The OOI Dashboard highlights significant geographic disparities in infant mortality, revealing how certain census tracts with high deprivation correlate with poor health outcomes. The dashboard supports targeted interventions by policymakers and healthcare providers to improve conditions in high-risk communities.
Joanna Almeida, 2022 et-al US [28]	Focus on racial/ethnic disparities in preterm birth (PTB), particularly between non-Latinx Black and White women in the US.	Commentary reviewing extant research on explanations for racial/ethnic gap in adverse birth outcomes such as PTB	Pregnancy Risk Assessment Monitoring System (PRAMS) to include a standardized measure of racial discrimination across all states to better assess its impact on maternal and infant health.	Preterm birth, infant mortality, and the effects of interpersonal racial discrimination on adverse birth outcomes	The study highlights the significant impact of racial discrimination on health inequities, particularly in preterm birth rates. It calls for PRAMS to adopt a validated, multi-item measure of discrimination to improve the understanding of racial/ethnic disparities in birth outcomes.
Serena Donati et-al, 2019, Italy [2]	Maternal deaths in eight Italian regions covering 73% of national births between 2013-2017.	Retrospective study conducted reviewed to assess quality of care and establish the cause and avoidability of the death.	The Italian Obstetric Surveillance System (ItOSS) is based on incident case reporting and confidential enquiries to assess maternal deaths, quality of care, and avoidability, involving both public and private health facilities.	Maternal mortality, with primary causes including obstetric haemorrhage, maternal sepsis, and hypertensive disorders of pregnancy	The study identified 106 maternal deaths over five years, with a maternal mortality rate of 7.56 per 100,000 live births. Approximately 39% of these deaths were deemed avoidable, highlighting issues such as delays in diagnosis, inadequate communication, and inappropriate care.
Caroline Diguisto et-al, 2022,	297,835 live births in Denmark, 301,169 in	Descriptive multicounty population-based study	Enhanced surveillance systems were used in all eight	Maternal mortality ratios up to 42 days post-pregnancy,	Maternal mortality varied significantly, with Norway and Denmark having the lowest ratios (2.7–3.4 per 100,000 live

Denmark, Finland, France, Italy, Netherlands, Norway, Slovakia, UK [25]	Finland, 2,435,583 in France, 1,281,986 in Italy, 856,572 in the Netherlands, 292,315 in Norway, 283,930 in Slovakia, and 2,261,090 in the UK (2013-2018)	of eight countries.	countries to document and review maternal deaths, ensuring standardization in identifying and classifying cases.	with leading causes including cardiovascular diseases, suicide, venous thromboembolism, and obstetric haemorrhage.	births) and Slovakia and the UK the highest (9.6–10.9 per 100,000). Vital statistics underestimated maternal mortality by 36% or more in most countries.
Lauren D. Arnold, et-al 2019 US [21]	Analysis of Pregnancy Risk Assessment Monitoring System (PRAMS) data (2012–2015), focusing on pregnant women across the United States (n = 130,161).	Cross-sectional, population-based analysis of PRAM between 2012 and 2015.	Pregnancy Risk Assessment Monitoring System (PRAMS) to include a standardized measure of racial discrimination across all states to better assess its impact on maternal and infant health	Maternal influenza vaccine uptake during pregnancy and healthcare provider recommendations, stratified by race/ethnicity.	Significant racial disparities were identified, with lower vaccine uptake among non-Hispanic Black women (39.1%) compared to non-Hispanic White women (49.8%).
Ann E. Sprague et-al, 2013, Canada [20]	Maternal-newborn populations across 109 hospitals in Ontario, where approximately 140,000 births occur annually.	Development of a clinical dashboard using key performance indicators (KPIs) based on data from the BORN Ontario system. Utilized surveys, data mapping, and feedback loops.	A real-time maternal-newborn dashboard with drill-down capacity for detailed analysis, integrating KPIs across six quality domains (e.g., accessibility, safety).	Episiotomy rates, Caesarean section rates, postpartum haemorrhage, maternal satisfaction, newborn screening, breastfeeding rates, and more.	Significant variation in maternal-newborn clinical practices across Ontario. The dashboard helped identify areas needing quality improvement and provided benchmarks for care.
Micheal Sun et-al 2022, US [22]	Included 18,459 patients from an urban academic medical centre in Chicago, who were tested for COVID-19 between January 2019 and October 2020.	Cross-sectional analysis using machine learning and natural language processing (NLP) to identify negative patient descriptors in electronic health records (EHRs).	No specific dashboard was created. Instead, the study used EHRs to analyse bias in patient documentation.	Focused on racial bias in patient documentation, particularly the use of negative descriptors, rather than maternal or neonatal outcomes.	Black patients had 2.54 times higher odds of having negative descriptors (e.g., non-compliant, agitated) in their medical notes compared to White patients, highlighting racial bias.
Kyle Wark, et-al, 2022, US [23]	Alaska Native and American Indian (ANAI) communities, involving 65,000 individuals served by the Southcentral Foundation.	Case study using stakeholder engagement through focus groups, steering committee meetings, and	Development of dashboards within EHRs to visualize SDOH data and track health outcomes for ANAI populations.	Improve maternal-child health outcomes by integrating SDOH data, but specific maternal and neonatal outcomes are not detailed.	Stakeholder engagement is crucial for designing culturally appropriate, non-stigmatizing SDOH data frameworks. This approach improves patient-provider relationships and care delivery.

		workgroups to integrate SDOH data into EHRs.			
Lisa Romero, et-al, 2022, US [24]	Populations served by federally qualified health centres, particularly racial and ethnic minorities, homeless individuals, and people in public housing.	Multistate quality improvement initiative using EHR data to develop a reusable public health analytics system addressing COVID-19 data challenges.	A public health registry designed to aggregate COVID-19-related data across health centres, including dashboards for public health surveillance and organizational planning.	Not applicable. The focus is on COVID-19 disparities and public health responses, not specific maternal or neonatal outcomes	Interoperability and data sharing challenges were significant barriers to real-time data analytics. The project improved data standardization and quality improvement efforts.
Marian Knight, et-al 2022, UK & Ireland [9]	Includes women who died during or up to one year after pregnancy between 2018 and 2020 in the UK and Ireland, focusing on maternal deaths and morbidity	Confidential Enquiries into maternal deaths, with multidisciplinary reviews of care, and comparison against guidelines and standards (e.g., NICE).	The report uses national surveillance data and confidential enquiry reviews to inform recommendations.	Maternal mortality, suicide, cardiovascular disease, mental health issues, hypertensive disorders, and diabetic ketoacidosis.	Maternal death rates rose, with Black women 3.7x more likely to die than White women. Thrombosis and thromboembolism remain leading causes, and maternal suicide has increased.
Marian Kinght et-al, 2016 UK & Ireland [7]	Women who died during or up to one year after pregnancy between 2009 and 2014, focusing on maternal deaths and morbidity in the UK and Ireland.	Confidential Enquiries into maternal deaths using surveillance data and reviews by multidisciplinary teams.	The report uses national surveillance data and confidential enquiry reviews to inform recommendations.	Maternal mortality, hypertensive disorders, cardiovascular disease, sepsis, thrombosis, and mental health outcomes, including maternal suicide.	Maternal deaths have not significantly decreased; cardiovascular disease remains the leading cause of maternal mortality. Hypertensive disorders have seen improved outcomes.
Gabrielle Hester, et-al 2018, United states [16]	Paediatric patients at Children's Minnesota with over 500,000 patient encounters per year, focusing on racial and ethnic health disparities.	Quality improvement (QI) methods. Data were pulled from electronic health records (EHR) and stratified by race, ethnicity, and language. Metrics were updated quarterly, and disparities were analyzed to inform targeted interventions.	A Paediatric Health Equity Dashboard (PHED) was designed to track disparities in outcomes such as asthma control, vaccinations, and appointment attendance, stratified by race and ethnicity.	Focus on pediatric outcomes such as well-controlled asthma, combo-10 vaccines, procedure-related pain control, and no-show appointments.	Significant disparities were found, with Black/African American children less likely to achieve well-controlled asthma or complete vaccinations compared to White children. Targeted interventions were implemented to address these gaps.

Appendix B: Search Strategy

Search Strategy

1 Electronic Health Records/ 25979

2 decision making, computer-assisted/ or decision support systems, clinical/ 11834

3 Medical Records Systems, Computerized/ 19143

4 dashboard*.tw,kw. 2319

5 "clinical decision support".kw. 1055

6 (Computer* adj3 health record*).tw,kw. 166

7 (computer* adj3 interface*).tw,kw. 7279

8 (computer* adj3 medical record*).tw,kw. 1524

9 electronic health record*.tw,kw. 26025

10 ((electronic* or computer* or data) adj3 (monitor* or visuali* or display*)).tw,kw. 35434

11 data display/ or informatics/ 8152

12 data display*.tw,kw. 1106

13 informatic*.tw,kw. 16473

14 or/1-13 132787

15 (Health* adj3 (inequalit* or discrimat* or disparit* or injust* or barrier* or unqual*)).tw,kw. 42883

16 Healthcare Disparities/ 21541

17 health inequities/ 232

18 15 or 16 or 17 59309

19 14 and 18 879

20 (Educat* or Race* or Racial or Social* or economic or religious or financ* or socioeconomic* or ethnic* or gender* or sex* or age* or disabilit* or bias or homophob* or xenophob* or residenc* or prejudice* or discrimin*).tw,kw. 6614365

21 Prejudice/ 25468

22 social discrimination/ or ageism/ or homophobia/ or perceived discrimination/ or racism/ or

sexism/ or xenophobia/ 11611

23 20 or 21 or 22 6625526

24 19 and 23 629

25 exp Pregnancy/ 983452

26 Obstetrics/ 24272

27 maternal health services/ or maternal-child health services/ or perinatal care/ or prenatal care/ 50376

28 matern*.tw,kw. 328267

29 Pregnanc*.tw,kw. 494392

30 Obstetric*.tw,kw. 113106

31 (perinatal* or peri-natal*).tw,kw. 86625

32 (pre-natal* or prenatal*).tw,kw. 116988

33 post natal*.tw,kw. 8534

34 labo?r*.tw,kw. 795004

35 birth*.tw,kw. 385253

36 exp Infant, Newborn/ 661912

37 (neonat* or infant* or newborn* or baby or babies).tw,kw. 810272

38 preterm*.tw,kw. 90044

39 obstetric labor, premature/ or premature birth/ 32230

40 "maternal-child health".kw. 132

41 or/25-40 2833494

42 24 and 41 98

43 19 and 41 114

Appendix C. Preferred Reporting Items for Systematic reviews and Meta-Analyses extension for Scoping Reviews (PRISMA-ScR) Checklist

SECTION	ITEM	PRISMA-ScR CHECKLIST ITEM	REPORTED ON PAGE #
**TITLE**			
Title	1	Identify the report as a scoping review.	1
**ABSTRACT**			
Structured summary	2	Provide a structured summary that includes (as applicable): background, objectives, eligibility criteria, sources of evidence, charting methods, results, and conclusions that relate to the review questions and objectives.	2
**INTRODUCTION**			
Rationale	3	Describe the rationale for the review in the context of what is already known. Explain why the review questions/objectives lend themselves to a scoping review approach.	3-4
Objectives	4	Provide an explicit statement of the questions and objectives being addressed with reference to their key elements (e.g., population or participants, concepts, and context) or other relevant key elements used to conceptualize the review questions and/or objectives.	4
**METHODS**			
Protocol and registration	5	Indicate whether a review protocol exists; state if and where it can be accessed (e.g., a Web address); and if available, provide registration information, including the registration number.	nil
Eligibility criteria	6	Specify characteristics of the sources of evidence used as eligibility criteria (e.g., years considered, language, and publication status), and provide a rationale.	4-5
Information sources*	7	Describe all information sources in the search (e.g., databases with dates of coverage and contact with authors to identify additional sources), as well as the date the most recent search was executed.	4
Search	8	Present the full electronic search strategy for at least 1 database, including any limits used, such that it could be repeated.	20

Selection of sources of evidence†	9	State the process for selecting sources of evidence (i.e., screening and eligibility) included in the scoping review.	6-7
Data charting process‡	10	Describe the methods of charting data from the included sources of evidence (e.g., calibrated forms or forms that have been tested by the team before their use, and whether data charting was done independently or in duplicate) and any processes for obtaining and confirming data from investigators.	6
Data items	11	List and define all variables for which data were sought and any assumptions and simplifications made.	6
Critical appraisal of individual sources of evidence§	12	If done, provide a rationale for conducting a critical appraisal of included sources of evidence; describe the methods used and how this information was used in any data synthesis (if appropriate).	nil
Synthesis of results	13	Describe the methods of handling and summarizing the data that were charted.	6
**RESULTS**			
Selection of sources of evidence	14	Give numbers of sources of evidence screened, assessed for eligibility, and included in the review, with reasons for exclusions at each stage, ideally using a flow diagram.	6-7 (See fig 1)
Characteristics of sources of evidence	15	For each source of evidence, present characteristics for which data were charted and provide the citations.	7-8
Critical appraisal within sources of evidence	16	If done, present data on critical appraisal of included sources of evidence (see item 12).	nil
Results of individual sources of evidence	17	For each included source of evidence, present the relevant data that were charted that relate to the review questions and objectives.	17-18
Synthesis of results	18	Summarize and/or present the charting results as they relate to the review questions and objectives.	8-11
**DISCUSSION**			
Summary of evidence	19	Summarize the main results (including an overview of concepts, themes, and types of evidence available), link to the review questions and objectives, and consider the relevance to key groups.	12-14
Limitations	20	Discuss the limitations of the scoping review process.	14-15

Conclusions	21	Provide a general interpretation of the results with respect to the review questions and objectives, as well as potential implications and/or next steps.	15
**FUNDING**			
Funding	22	Describe sources of funding for the included sources of evidence, as well as sources of funding for the scoping review. Describe the role of the funders of the scoping review.	15

Appendix D: Supplementary Table of Excluded Studies with Reasons

Authors	Reasons for Exclusion
Davidson, Christina; et-al 2022 [45]	Evaluates quality improvement initiatives, not the use of dashboards.
Dirksen, Jakob et-al 2022 [46]	Examines socioeconomic deprivation status, unrelated to dashboards or maternal outcomes.
Doll, Kemi M. et-al 2022 [47]	Develops an algorithm for symptom severity, not related to inequality dashboards.
Fabbro, M. R. C. et-al 2022 [48]	Analyses antenatal care impact, not implementation of health inequality dashboards.
Partha Saha et-al 2019 [49]	While it addresses inequities, it does so through location-specific policies and interventions rather than evaluating or addressing the implementation, use, or challenges of health inequality dashboards specifically targeting maternal and neonatal outcomes
Sanhueza, A et-al 2002 [50]	Supports regional equity strategies, unrelated to health inequality dashboards.
Sun, Michael et-al 2022	Analyses racial bias in medical records, not health dashboards.
GOMEZ, HELEN et-al 2021[51]	Describes telehealth usage in postpartum care, not dashboards for inequalities.
Jean-Francois, et-al 2021[52]	Reviews health IT tools for racial disparities, not dashboard implementation.
Pandey, A. R. et-al 2021 [53]	Analyses Nepal's health progress, unrelated to health inequality dashboards.
Alexandra ZINGG et-al 2021[54]	Explores the use of digital health technologies for maternal health with no reference to dashboards
Sadovsky, Yoel et-al 2020 [55]	Discusses technological advances in pregnancy, unrelated to dashboards.
Daniel J Miklin et-al 2019 [56]	The study examines the perceptions, access issues, and technology gaps in using EHR portals among adolescents, which are unrelated to the implementation, use, or challenges of health inequality dashboards
Bingham, D. et-al 2019 [57]	Uses quality improvement strategies, unrelated to dashboard use.
Cottrell, E.; et-al 2019 [58]	Assesses ACA impacts on women's healthcare, not dashboard implementation.
Murphy, V. E.; et-al 2019	Examines NICU admissions for asthma-related pregnancies, unrelated to dashboards.
Schwarz, L. et-al 2019 [59]	Investigates birth weight disparities due to pollution, not dashboard use.
Guo, Yuqing et-al 2018 [60]	Explores electronic health record access in underserved populations, not dashboards.
Ukoha, Erinma et-al 2018 [61]	Focuses on disparities in patient portal use during pregnancy, not dashboards.
Klumpner, T. T et-al 2017 [62]	Reviews big data applications in anaesthesiology, not related to inequality dashboards.
